# Discovering the Molecular Landscape of Meningioma: The Struggle to Find New Therapeutic Targets

**DOI:** 10.3390/diagnostics11101852

**Published:** 2021-10-08

**Authors:** Ilaria Maggio, Enrico Franceschi, Vincenzo Di Nunno, Lidia Gatto, Alicia Tosoni, Daniele Angelini, Stefania Bartolini, Raffaele Lodi, Alba Ariela Brandes

**Affiliations:** 1 Medical Oncology Department, Azienda USL, Via Altura n. 3, 40139 Bologna, Italy; ila.mag.88@gmail.com (I.M.); dinunnovincenzo88@gmail.com (V.D.N.); lidia.gatto83@gmail.com (L.G.); 2 Nervous System Medical Oncology Department, IRCSS Istituto di Scienze Neurologiche di Bologna, 40139 Bologna, Italy; a.tosoni@ausl.bologna.it (A.T.); daniele.angelini@ausl.bologna.it (D.A.); stefania.bartolini@ausl.bologna.it (S.B.); alba.brandes@yahoo.it (A.A.B.); 3 IRCCS Istituto delle Scienze Neurologiche di Bologna, 40139 Bologna, Italy; raffaele.lodi@isnb.it or

**Keywords:** meningioma, NF2, SMO, DNA methylation, molecular alterations

## Abstract

Meningiomas are the most common primary CNS tumors. They are usually benign but can present aggressive behavior in about 20% of cases. The genetic landscape of meningioma is characterized by the presence (in about 60% of cases) or absence of NF2 mutation. Low-grade meningiomas can also present other genetic alterations, particularly affecting SMO, TRAF7, KLF4 AKT1 and PI3KCA. In higher grade meningiomas, mutations of TERT promoter and deletion of CDKN2A/B seem to have a prognostic value. Furthermore, other genetic alterations have been identified, such as BAP1, DMD and PBRM1. Different subgroups of DNA methylation appear to be correlated with prognosis. In this review, we explored the genetic landscape of meningiomas and the possible therapeutic implications.

## 1. Introduction

Meningiomas are central nervous system (CNS) tumors that originate from arachnoid cells in the inner surface of the dura. With an incidence of 7.86 cases per 100,000 people per year [1], meningiomas represent the most frequent CNS tumor, accounting for about 36% of all cases and 53% of benign lesions [1]. They are often benign and mostly diagnosed incidentally [2].

Many risk factors have been associated with development of meningioma. Among these, diabetes mellitus, arterial hypertension, smoking, and mobile phone use have been investigated, but results are inconclusive [3,4]. A recognized risk factor is ionizing radiation to the skull, with an increased risk of developing meningiomas six-fold to ten-fold greater [5,6]. Meningiomas that originated after irradiation are often multiple and present aggressive behavior [7]. In addition, sex hormones have been investigated as risk factors since there is a higher incidence of meningiomas in post-puberal women (2:1 versus men), with a ratio of about 3:1 in the reproductive period, but there is no definitive evidence [4,8,9,10,11,12].

Meningiomas can also be genetically determined and are present in several genetic syndromes such as: neurofibromatosis type 2 (NF2), which is most frequently associated with meningiomas, Gorlin, Li Fraumeni, Cowden, von Hippel–Lindau, and multiple endocrine neoplasia type 1 (MEN1). In these genetic syndromes, meningiomas are often multiple and arise in children [13].

Ten-year overall survival (OS) of benign meningiomas is about 81.4%, while for malignant ones, it is 57.1%, with about 53% for grade II and 0% for grade III. The recurrence rate for grade II is about 50%, and for grade III, it is 90% [14]. Disease progression is defined not only by the growth of the residual tumor, but also by the transformation into a higher-grade tumor.

In the 2016 WHO classification, meningiomas are distinct in different grades [15], as reported in Table 1.

Different grades present distinct histological and clinical features, but molecular alterations are not included due to the scant evidence about this topic [16].

We performed a review of the literature to summarize the current knowledge on meningiomas with a specific insight on the molecular alterations and possible therapeutic strategies. We carried out research on Pubmed/Medline, Cochrane library and Scopus using the keywords “meningioma” or “anaplastic” or “atypical” or “CNS” or “brain tumor” and we selected pivotal studies, while considering the quality of the study, how it was conducted, and the strength of the results.

## 2. Molecular Alterations

Meningiomas present immunohistochemical markers that are epithelial membrane antigens, in particular: somatostatin receptor 2A (SSTR2A) and progesterone receptor in 70–80% of cases, and estrogen receptor in about 5–30% of cases [17,18].

New genomic analysis techniques such as whole genomic sequencing (WGS), transcriptome analysis, whole exome sequencing (WES), DNA methylation assay, and chromatin immunoprecipitation sequencing allowed the characterization of the mutational landscape of meningiomas, with consequent identification of possible druggable targets [19].

### 2.1. Genomic Analysis Alterations

A common molecular alteration in meningiomas is 22q12 chromosome deletion that encodes for the tumor suppressor gene Merlin (also called neurofibromin 2, NF2), present in about half of all tumors [20,21]. NF2 mutation predispose to the development of meningiomas, and it seems to be associated with fibroblastic/transitional meningiomas [20,22]. NF2 inactivation gives to the tumor a genomic instability and a peculiar multiple localization in the hemispheres [23]. It is not yet well understood how the inactivation of NF2 can guide the development of meningiomas. Some hypotheses suggest that Merlin can inhibit cell proliferation with a contact-dependent regulation including Hippo, Notch and Patched pathway [24] and that it can activate the mammalian target of rapamycin (mTOR) pathway during tumor development [25,26]. The high frequency of this mutation can create a subdivision of meningiomas into NF2 and non-NF2 mutated.

Four mutually exclusive pathways that can guide the development of meningiomas have been identified through genomic analysis [27,28].

#### 2.1.1. Increased Hedgehog Signaling (through Mutation of SMO, SUFU or PRKAR1A)

Smoothened, frizzled class receptor (SMO) is a gene that encodes the transmembrane receptor protein smoothened homolog. This receptor is responsible for the transmission of signals from Hedgehog ligands to cells, leading to constitutive activation of SMO. SMO and v-akt murine thymoma viral oncogene homolog 1 (AKT1) can lead to the activation of PI3K–AKT–mTOR pathway; it is relatively common and is associated with meningiomas localized in the skull base and with genomic stability [20,29].

AKT1 mutations seems to be associated with grade I meningothelial meningiomas of the spine and skull base [20,30]. Somatic mutations at codon 17 are associated with higher risk of recurrence [30].

Suppressor of fused (SUFU) and patched 1 mutations can activate the SHH pathway, and somatic mutations of SUFU are present in 1% of sporadic meningiomas [30]. These alterations are commonly found in Gorlin syndrome [31].

#### 2.1.2. TRAF7 (through KLF4 Mutation or PI3K Pathway Activation)

TRAF7 mutations is present in about 25% of WHO grade I and II meningiomas. The most frequent alteration is in the WD40 domain, which is involved in the regulation of JUN N-terminal kinase (JNK) and p38 mitogen-activated protein kinase (MAPK) signaling [23,27,32]. TRAF7 mutation can be associated with AKT1, Krupple-like factor 4 (KFL4) or phosphatidylinositol-4,5- bisphosphate 3-kinase catalytic subunit alpha (PIK3CA) alteration, but it is mutually exclusive with NF2 and SMO mutations [30].

PIK3CA mutations are mutually exclusive with AKT1, SMO and NF2, and they can rarely be co-present to TRAF7 mutation [30].

KLF4 K409Q missense mutations are present in up to 50% of NF2 nonmutated meningiomas, often co-present with TRAF7 mutations, and lead to upregulation of HIF-1a pathway. Furthermore, KLF4 and TRAF7 seem to be associated with secretory meningiomas with more aggressive behavior due to increased swelling of the brain [23,33].

#### 2.1.3. POLR2A Mutations

Mutations on two hotspots of RNA polymerase II subunit A (POLR2A) are associated with meningothelial histology, usually localized in the tuberculum sellae [28].

#### 2.1.4. Other Rarer Mutations

Knowledge of molecular alterations of WHO grade III meningiomas is limited because of the rarity of the disease. In rhabdoid meningiomas, the frequent inactivation of breast cancer-associated protein 1 (BAP1) has been identified, and it seems to be associated with early tumor recurrence [34]. In papillary meningiomas, the biallelic inactivation of polybromo 1 (PBRM1) gene has been described, which can overlap with BAP1 mutations, but its role is not yet understood [35].

In atypical meningiomas, the upregulation of enhancer of zeste homolog 2 (EZH2) that interacts with epigenetic mechanisms can be present [36,37], and it seems to be a marker of aggressiveness and higher grade [38]. In particular, the expression of EZH2 and H3K27me3 negativity has been associated with shorter progression-free survival (PFS), thus highlighting a potential prognostic role for these biomarkers [38].

Duchenne muscular dystrophy (DMD) gene encodes for the protein dystrophin and its mutation has also been pointed out in meningiomas. Somatic DMD deletions seem to be associated with worse prognosis [39].

Mutations of KDM5C (Lysine Demethylase 5C), KDM6A or Somatic SWI/SNF-related matrix associated actin-dependent regulation of chromatin subfamily B member 1 protein (SMARCB1) can lead to epigenetic modifications that are present in about 10% of non-NF2 meningiomas [20].

Furthermore, telomerase reverse transcriptase (TERT) promoter mutations have never been detected in de novo atypical meningiomas but are present in secondary atypical meningiomas progressed from lower-grade primary tumors [40,41]. These findings highlight the role of TERT promoter alteration as a possible prognostic marker of recurrence and survival. Several studies have shown that TERT promoter mutation in meningiomas is associated with worse prognosis and shorter overall survival independent of WHO grade [42,43,44,45].

Mixed-lineage leukemia 1/Lysine (K)-specific MethylTransferase 2 (MLL/KMT2) mutation has been identified in WHO grade 2 chordoid meningioma, associated with NF2 mutations and with worse prognosis [46].

Cyclin-dependent kinase Inhibitor 2A/B (CDKN2A/B) plays an important role in cell cycle regulation, acting as a tumor suppressor. It has been found to be alternated in meningiomas, and its alteration can lead to the progression from grade 2 to grade 3 meningioma [38,47]. Somatic mutations and homozygous losses of CDKN2A and CDKN2B have been identified in anaplastic meningiomas [48,49,50]. A retrospective analysis of 67 atypical meningiomas showed that overexpression of p16, CDK6, and pRB protein predicted recurrence [49]. Moreover, a next-generation sequencing analysis of 17 recurrent and 13 non-recurrent meningiomas identified three CDKN2A alteration (p.Ala148Thr) mutation, whole homozygous or heterozygous gene loss, or promotor methylation (>8) strongly correlated with recurrence and a Ki67 labeling index > 7% [51].

The main molecular alterations are provided in Table 2.

### 2.2. Copy Number Alterations

Overall, copy number aberrations (CNA) have been associated with risk of recurrence in patients with resected atypical meningiomas, and it can represent a marker to help in the decision-making process on whether to start adjuvant radiotherapy [30]. The most frequent CNAs are 22q deletion, 1p, 14q, 9p loss, which are associated with higher grade meningiomas [53], and 6q, 10q, and 18q loss, or the gain of 1q, 9q, 12q, 15q, 17q, 19q, 20q and 5 chromosomes [36,54]. In recurrent tumors, CNAs overlap in about 75% the primary tumors, suggesting that they may play a key role in tumor development [36,54].

In addition to NF2, another gene located on chromosome 22q that can be altered is CHEK2, which has been described in NF2 mutated meningiomas. It is a tumor suppressor involved in DNA repair, and its mutations seem to contribute to genome instability [52].

### 2.3. Methylation Profiles

Through analysis of DNA methylation profiles, six subclasses of meningiomas have been identified, characterized by different histology, cytogenetic, mutational and gene expression patterns, and associated with different risk of recurrence [55]:

- group A: four methylation classes (MC benign (ben)-1 (*n* = 113), MC ben-2 (*n* = 118), and MC ben-3 (*n* = 73), MC intermediate (int)-A (*n* = 105));
- group B: two methylation classes (MC int-B (*n* = 47), MC malignant (mal) (*n* = 41)).

This classification is superior to WHO grading in terms of predicting PFS. In particular, MC intermediate patients with WHO grade I meningiomas resulted in a worse prognosis than patients characterized as grade I only by histology. Furthermore, grade I MC intermediate patients seem to have similar prognoses to WHO grade II ones, while grade II MC benign meningiomas had a better prognosis than WHO grade II ones.

Olar and colleagues identified 64 CpG loci methylation predictors correlated with different recurrence risks [56]. In this study, two subgroups of patients were identified: a prognostically unfavorable and a prognostically favorable molecular methylation subgroup. The prognostically unfavorable subgroup presented a higher proportion of copy number aberrations, including losses of 1p, 6q, 14q and 18q, and a gain of 1q, all associated with poor outcomes, with 185 hypermethylated CpG loci and a median RFS of 12.07 years (range 0.31–17.61 years). The favorable subgroup presented 98 hypermethylated CpG loci and had a median recurrence-free survival (RFS) not reached (range 0.27–16.6 years).

A nomogram integrating clinical factors and DNA methylation profiles has been developed to predict early meningioma recurrence. The 5-year methylome predictor subdivided patients into lower and higher risk groups with different FRS in all three validation cohorts of the study. In particular, patients in the higher risk group had a median RFS of 2.1, 8.1, and 4.2 years in the first, second, and third validation cohorts, respectively, compared with patients in the lower risk groups, which presented a median RFS of unreached in the first and second validation cohorts and of 7.2 years in the third validation cohort while higher risk groups had a median RFS of 2.1, 8.1, and 4.2 years in the three validation cohorts, respectively [57].

Molecular analysis allowed to clarify the biological landscape of two other types of meningiomas, with possible therapeutic implications: radiation-induced and progestin-associated meningiomas [58,59]. Progestine-associated meningiomas, which are generally grade 1 and 2, present NF2 alteration only in 7.5% of cases, while a PIK3CA mutation can be found in 35% of cases [59,60]. Otherwise, radiation-induced meningiomas often present NF2 alterations and deficit of DNA double strand repair genes [61].

The increasing knowledge of the molecular landscape of meningiomas has allowed individuation of prognostic and predictive markers that can guide therapeutic decision-making processes and timing of follow-up. Nonetheless, molecular features need to be integrated with histopathologic and clinical characteristics to better help predict the risk of recurrence.

## 3. Treatment Options

The choice of the more appropriate treatment approach depends on growth rate, grade, localization and symptoms of meningioma.

### 3.1. Surgery

Surgery is the cornerstone treatment for growing and symptomatic meningiomas, while for asymptomatic ones, radiological surveillance can be considered [62]. It should be as radical as possible, and the radicality of resection is measured by the Simpson grade, reported in Table 3 [63]. The risk of recurrence is higher if residual disease is present, and this can happen if the localization of meningioma limits the surgical approach. After gross total resection, the 5-year recurrence rate is 7–23% for grade I meningiomas, 50–55% for grade II and 72–78% for grade III ones [14].

### 3.2. Radiotherapy

Meningiomas not suitable for surgery can be irradiated, but radiotherapy is mainly used in an adjuvant setting after surgical resection or to treat recurrent disease. Currently, there is no definitive evidence on timing, dose and fractioning of radiotherapy in meningiomas because of the lack of randomized controlled trials. It is not indicated in radically resected WHO grade I meningiomas, but it can be proposed in cases of residual disease [13]. In grade II meningiomas, it still plays a controversial role, but it can be considered in cases of incomplete resection [64]. In grade III meningiomas, adjuvant radiotherapy should be used to improve local control [65].

### 3.3. Systemic Treatment

The use of systemic treatment is largely experimental since several drugs have been tested with limited results. Currently, there is not a standard of care treatment or definitive evidence on preferable therapies and their sequence. Enrollment in clinical trial is highly recommended [66].

Some prospective and retrospective studies investigated different types of treatments, including target therapies such as vascular endothelial growth factor (VEGF)/receptor (VEGFR) inhibitors [67,68,69,70] or epidermal growth factor receptor (EGFR) inhibitors [71] and chemotherapies such as as hydroxyurea [72,73,74], temozolomide [75], irinotecan [76], and trabectedin [77,78]. These latter compounds are not recommended due to their scant efficacy. Molecular alteration presented by meningiomas suggests the investigation of targeted therapy agents. Meningiomas are highly vascularized tumors and present an upregulated expression of VEGF [79], supporting the study of anti-angiogenic agents in phase II trials [80]. Among these, there are monoclonal antibody against VEGF bevacizumab [68,69,70,81,82,83], the tyrosine kinase inhibitor (TKI) directed against VEGFR sunitinib, the TKI against platelet-derived growth factor receptor-beta (PDGFR-beta) c-kit, VEGFR-2 vatalanib [84], the TKI against PDGFR c-kit, and BCR-ABL imatinib [85].

The inactivation of NF2 and its interaction with the mTOR pathway, with a negative regulation of mTORC1, which results in overexpression and a positive regulation of mTORC2 [86,87,88], supported the investigation of mTOR inhibitors. Furthermore, KLF4 K409Q mutation, evidenced in 10–14% of meningiomas and found in combination with TRAF7 mutations in the secretory subtype, upregulates hypoxia-inducible factor-1a (HIF-1a) pathway, thus creating a state of cellular hypoxia with the subsequent activation of mTOR pathway. Consequently, mTOR inhibitors have been experimented as possible treatment strategies [89].

Everolimus has been tested in combination with octreotide [90] and with bevacizumab [70]. In the phase II CEVOREM trial, the combination of everolimus 10 mg daily and octreotide 30 mg on day 1 was investigated in 20 patients with recurrent progressive meningiomas ineligible for further surgery or radiotherapy (WHO grade I, II, or III). The experimental combination showed clinical and radiological activity. Everolimus 10 mg daily was also investigated in a phase II trial in combination with bevacizumab (10 mg/kg IV days 1 and 15) in 17 patients with recurrent meningioma (WHO grade I, II, or III) following standard treatments with surgical resection and radiotherapy. The combination resulted to be well tolerated and obtained stable disease in 88% of patients.

Vistusertib (AZD2014), an inhibitor of mTORC1 and mTORC2, is being testing in NCT03071874 trial, based on promising results of preclinical studies, in both recurrent and progressive meningiomas [91]. Activating mutations of PIK3-AKT-mTOR pathway is being evaluated as a therapeutic target in a phase II trial in progressive refractory meningiomas investigating the PI3K inhibitor alpelisib in combination with the MEK inhibitor trametinib (NCT03631953 trial) [27].

Dasatinib, an inhibitor of Src kinases, erythropoietin-producing hepatocellular receptor tyrosine kinases (EPH RTKs) and c-KIT, has been tested in glioblastoma [92], and it also showed an inhibition of growth in NF2-deficient meningiomas when associated with dual mTORC1/2 inhibitor [93].

The alteration of AKT1 can be targeted by AKT inhibitors such as capivasertinib (AZD5363). A patient with meningotheliomatous meningioma presenting AKT1E17K mutation, who progressed to surgery, radiotherapy and two previous lines of systemic therapies, was treated with capivasertinib with a durable disease control, thus suggesting this molecular alteration may be a potential therapeutic target [94].

Preclinical studies also showed the sensibility of NF2-mutated meningiomas to focal adhesion kinase (FAK) inhibition, which seem to have a synthetic lethal relationship with NF2 loss [95]. On the basis of this finding, a FAK inhibitor (GSK2256098) has been tested in a phase II trial (Alliance A071401, NCT02523014) in recurrent or progressive meningiomas (WHO I-III grade) [96]. The experimental drug resulted to improve 6-months PFS (one of the primary endpoints) and to be well tolerated, thus supporting further investigation of this treatment. The same FAK inhibitor (GSK2256098) is also being tested in a two-arm phase II trial (NCT02523014) in progressive meningiomas harboring NF2 mutations. In the second arm of this trial, patients with SMO mutations received an SMO and PTCH1 inhibitor (vismodegib).

BAP1-mutated meningiomas are associated with DNA damage repair defects [34]. In this subgroup, Poly ADP ribose polymerase (PARP) inhibitors are being investigated [97]. Furthermore, a phase II study (NCT02860286) is evaluating Tazemetostat, an EZH2 inhibitor, in tumors with BAP1 loss of function.

CDK inhibitors are another class of drugs being tested in meningiomas, given their role in suppressing tumor cell survival by depleting proteins such as MCL-1 and c-MYC. Classifying patients based on the genomic methylation profiling [55], the oral CDK inhibitor TG02 has been tested in meningioma cell cultures [98]. Another CDK inhibitor, ribociclib, has been tested in vivo, demonstrating to achieve pharmacologically active concentrations in aggressive meningioma tissue [99].

In progestin-associated meningiomas, the anti-progesterone drug mifepristone is being tested in a phase III trial against placebo in unresectable meningiomas (NCT03015701). In radiation-induced meningiomas, the alteration of DNA double strand break repair genes through the homologous recombination repair system could suggest the investigation of PARP inhibitors [58,100].

The identification of the expression of somatostatin receptor (SSTR) subtype 2 by [101] meningiomas led to investigating the role somatostatin analogs. In vitro studies showed that octreotide, a somatostatin analog, presented an antiproliferative effect with reduced tumor growth, but its effect did not result in tumor shrinkage. Further trials confirmed the absence of clinical effect of these compounds in meningioma [102].

## 4. Conclusions

Novel molecular characterization techniques allow understanding of the biology underlying the development and progression of meningiomas. Several molecular alterations have been shown, with prognostic and therapeutic implications. Novel therapeutic approaches are being tested to target peculiar mutations. This biological evidence and the new treatments under investigation could possibly change the approach to meningioma relapse or refractory to surgery and radiotherapy.

## Figures and Tables

**Table 1:** Meningiomas grading and frequency [15].

WHO Grade	Description	Frequency (%)
Grade I	No brain invasion Mitotic rate < 4 per 10 HPFs	80–85
Grade II Atypical	Brain invasion Or Mitotic rate 4–19 per 10 HPFs Or ≥3 or 5 specific histological characteristics: - spontaneous or geographic necrosis - patternless sheet-like growth - prominent nucleoli - high cellularity - small cells with high n:c ratio	15–20
Grade III Anaplastic Malignant	Mitotic rate > 20 per 10 HPFs Or Papillary or rhabdoid histologies	1–2

HPF: high-power field.

**Table 2:** Molecular alteration in meningiomas and correlated clinical characteristics.

Gene	Molecular Alteration	Pathway	Tumor Histology and Grade	Grade	Tumor Localization	Frequency
NF2 [20,21,22,23,24,25,26]	- 22q12 chromosome deletion - Genomic instability	Activation of PI3K–AKT–mTOR pathway and Hippo pathway	Fibrous and transitional	I–III	Hemispheres, often bilateral	40–60%
SMO [20,29]	- Leu412Pheand Trp535Leu mutations - Genomic stability	Activation of PI3K–AKT–mTOR and SHH pathway	Meningothelial	I	Skull base	1–5%
AKT1 [20,30]	p.Glu17Lys mutation	Activation of PI3K–AKT–mTOR pathway	Meningothelial	I	Spine and skull base	7–12%
SUFU [30,31]	Locus 10q24.32	SHH pathway	NA	NA	NA	1%
TRAF7 [23,27,30,32]	WD40 domains mutations	JUN N-terminal kinase (JNK) and p38 mitogen-activated protein kinase (MAPK) signaling	Secretory	I > II, III	Anterior and middle medial skull base	15–26%
PIK3CA [30]	Locus 3q26.32, especially in H1047R, E542K and E545K	Activation of PI3K–AKT–mTOR pathway	Meningothelial, transitional	I > II, III	Skull base	4–7%
KLF4 [23,33]	Locus 9p31	Activation of PI3K–AKT–mTOR pathway	Secretory	I	Middle and lateral skull base	9–12% Up to half of NF2 nonmutated meningiomas
POLAR2 [28]	- p.Gln403Lys mutation - p.Leu438_His439del	Transcription	Meningothelial	I	Tuberculum sellae	6%
BAP1 [34,35]	Multiple mutations	DNA repair	Rhabdoid, papillary	II, III	Convexity	<1%
PBRM1 [35]	Locus 3p21.1	Chromatin remodeling	Papillary, rhabdoid	NA	NA	2.8%
Epigenetic alterations [20,36,37,38]	Mutations in KDM5C, KDM6A, SMARCB1, EZH2	Chromatin remodeling	Multiple pathogenic variant EZH2: High grade	NA	NA	10% of non-NF2 meningiomas
DMD [39]	Locus Xp21.1	Cytoskeleton	NA	II, III	NA	NA
CDKNA2A/B [38,47,48,49,50]	Somatic mutations and homozygous losses	Cell cycle regulation	NA	II, III	NA	<5%
TERTp [40,41,42,43,44]	Locus sp15.33 in C228T and C250T	Telomerase activity	NA	I-III	NA	4.7–15.4%
CHECK2 [52]	Locus 22q12.1	Cell cycle regulation	NA	I	NA	Rare
FAK [19]	Locus 8q24.3	Cell motility	NA	NA	NA	Rare

NA: Not available.

**Table 3:** Simpson grade [63].

**1**	Complete resection, with dural and bone resection
**2**	Gross total resection with dural coagulation
**3**	Macroscopic resection, without dural excision or coagulation
**4**	Subtotal resection
**5**	Biopsy

## Data Availability

Data sharing not applicable.
